# A data analysis framework for biomedical big data: Application on mesoderm differentiation of human pluripotent stem cells

**DOI:** 10.1371/journal.pone.0179613

**Published:** 2017-06-27

**Authors:** Benjamin Ulfenborg, Alexander Karlsson, Maria Riveiro, Caroline Améen, Karolina Åkesson, Christian X. Andersson, Peter Sartipy, Jane Synnergren

**Affiliations:** 1 School of Bioscience, University of Skövde, Skövde, Sweden; 2 School of Informatics, University of Skövde, Skövde, Sweden; 3 Takara Bio Europe AB, Gothenburg, Sweden; 4 Cardiovascular and Metabolic Disease Global Medicines Development Unit, AstraZeneca, Gothenburg Mölndal, Sweden; Leiden University Medical Center, NETHERLANDS

## Abstract

The development of high-throughput biomolecular technologies has resulted in generation of vast omics data at an unprecedented rate. This is transforming biomedical research into a big data discipline, where the main challenges relate to the analysis and interpretation of data into new biological knowledge. The aim of this study was to develop a framework for biomedical big data analytics, and apply it for analyzing transcriptomics time series data from early differentiation of human pluripotent stem cells towards the mesoderm and cardiac lineages. To this end, transcriptome profiling by microarray was performed on differentiating human pluripotent stem cells sampled at eleven consecutive days. The gene expression data was analyzed using the five-stage analysis framework proposed in this study, including data preparation, exploratory data analysis, confirmatory analysis, biological knowledge discovery, and visualization of the results. Clustering analysis revealed several distinct expression profiles during differentiation. Genes with an early transient response were strongly related to embryonic- and mesendoderm development, for example *CER1* and *NODAL*. Pluripotency genes, such as *NANOG* and *SOX2*, exhibited substantial downregulation shortly after onset of differentiation. Rapid induction of genes related to metal ion response, cardiac tissue development, and muscle contraction were observed around day five and six. Several transcription factors were identified as potential regulators of these processes, e.g. *POU1F1*, *TCF4* and *TBP* for muscle contraction genes. Pathway analysis revealed temporal activity of several signaling pathways, for example the inhibition of WNT signaling on day 2 and its reactivation on day 4. This study provides a comprehensive characterization of biological events and key regulators of the early differentiation of human pluripotent stem cells towards the mesoderm and cardiac lineages. The proposed analysis framework can be used to structure data analysis in future research, both in stem cell differentiation, and more generally, in biomedical big data analytics.

## Introduction

The recent development of novel high-throughput molecular technologies has rendered the possibility to rapidly generate vast amounts of biomedical data at reasonable costs. Large-scale omics data is now widely available in the fields of transcriptomics, proteomics, metabolomics, and interactomics. This biomedical big data (BBD) is characterized by its size and complexity and has in some aspects transformed biomedical research into a data-driven discipline [1]. The bottleneck has now shifted from high costs for generation of the data to challenges related to the analysis and interpretation of the data into meaningful biological knowledge [2,3].

Human pluripotent stem cells (hPSCs) have the capabilities of self-renewal and pluripotency, and they can in principle differentiate into any cell types in the body. Thus, hPSCs is a promising source of human specialized cells for use in many different applications such as toxicity testing, drug development, and regenerative medicine [4,5]. However, to fully make use of this unique cell type and develop efficient and reproducible differentiation protocols, more knowledge is needed about regulatory mechanisms and molecular pathways important to efficiently direct the differentiation towards specific functional cell types [6]. A common strategy for characterization of the regulatory mechanisms underlying stem cell differentiation is time series transcriptome profiling experiments. High-throughput technology such as microarrays are used to measure global gene expression over time, usually followed by clustering analysis to identify groups of genes with similar expression profiles [7,8].

A recent high-resolution transcriptomic characterization of hESCs undergoing differentiation towards the cardiac lineage was provided by Piccini *et al*. [9]. Global expression profiling was carried out at nine discrete time points to highlight temporal changes in many known cardiac markers. In addition, differentiation factor withdrawal time-courses were carried out to assess the importance of different signaling pathways. Another study by van de Berg *et al*. [10] identified eight clusters of genes with similar profiles across four time points, and performed enrichment analysis to investigate significant biological processes. Examples of processes identified as enriched include cell cycle regulation, development, and muscle organization. Time series gene expression profiling has also been performed to explore the biological mechanisms involved in adipose stem cell (ASC) differentiation. Satish *et al*. [11] performed global expression profiling on six clinical samples at three times points to identify genes responding to adipocyte lineage differentiation. Genes with differential expression between time points were analyzed with Ingenuity Pathway analysis software and several significant pathways were reported. In a study by Yang *et al*. [12], miRNA expression was measured in ASC before and after induction of chondrogenic differentiation. The authors found 20 differentially expressed miRNAs and performed clustering analysis, but no detailed follow up analysis of the clustered miRNAs. Although many valuable biological insights are provided by these studies, given the high-throughput nature of the data, more comprehensive data analysis has the potential to deliver even more knowledge about the regulatory mechanisms behind differentiation and identify transcription factors in control of these processes.

To address the challenges in BBD analytics and knowledge discovery, frameworks for analyzing the data are needed [13]. A framework in this context is a sequential process that allow researchers to move from unprocessed BBD to biological knowledge. Current studies commonly employ a large number of analysis methodologies combined in different ways, not always in a systematic manner. This may give specific biological insights, but omit other potential findings, and contribute to non-transparent analysis protocols. A framework on the other hand could ensure that data analysis is carried out in a comprehensive way, to extract as much biological knowledge as possible. In addition, such a framework would promote transparent and reproducible analysis protocols, and allow tracking of the provenance of data and results.

A key challenge in BBD analytics is the discovery of patterns in data through exploratory analysis. The basic idea of emphasizing the exploratory aspect within data analysis dates back to Tukey’s seminal work about “Exploratory Data Analysis” (EDA) [14] where he stress the significance of “[…] uncover indications […] for confirmatory data analysis […]” and that exploration of the data should be the “foundation stone” for any data analysis. One of the main goals of exploratory data analysis is to reveal different structures of the data and one of the most fundamental structures are based on similarity in the form of clusters, i.e., data items (e.g. genes) that share common properties. Genes with similar profiles may be functionally related and are therefore relevant to analyze as a group, to identify common functions and regulatory mechanisms. By investigating the functions of genes that show altered expression over time, it is possible to shed light on the biological processes and pathways involved in e.g. cellular differentiation [15].

Several clustering algorithms with different characteristics have been developed over the past decades [16–18]. Algorithms suitable for clustering of gene expression data can be classified based on different properties, for instance, type of data representation, relationship between clusters, distribution of the data, etc. [19]. Two widely used algorithms for analysis of gene expression data are k-means [20] and hierarchical clustering [18]. K-means is a randomized algorithm that generates cluster centers and assigns data items to the nearest cluster center; then the location of the centers is changed to minimize the sum of squared distances between items and their closest cluster centers [19]. Hierarchical clustering algorithms generate dendrograms that show relationships of objects and clusters as hierarchies. One challenge associated to both these methods is the specification of the number of clusters, k-means requires specification of the number of clusters before they are generated, while hierarchical clustering needs to select at which level the dendrogram should be cut. The choice of algorithm and its parameters should not be made arbitrarily, but be adapted to the data to uncover the most relevant structures.

The aims of the present study were to develop a data analysis framework for BBD and highlight the most important events and regulators during early differentiation of hPSCs towards the mesoderm lineage. To this end, global expression profiling of differentiating hPSCs was performed at 11 time points and the data subjected to the novel analysis framework proposed here. Despite some cellular heterogeneity in the collected cell samples, the analysis framework successfully identified both known and unknown biological processes, signaling pathways and transcription regulators that likely are highly involved in mesoderm and early cardiac differentiation.

## Materials and methods

### Cell lines and culturing of cells

Human embryonic stem cells (hESCs) from Cellartis^®^ hESC line SA121 (Takara-Clontech) were differentiated via the mesoderm germ layer towards the cardiac phenotype. The differentiation was initiated by adding BMP2/4, bFGF, Activin A, and BIO to the CM10 base medium. At day 3, the cells were detached with Collagenase IV and embryoid body (EB) formation was performed using forced aggregation [21] in a spin EB medium based on CM20 and supplemented with vEGF and small molecules targeting Wnt-, BMP- and TGF-signaling. The cell suspension was placed in 96 well plates, 200ul/well, and centrifuged 5 min at 400 g. At day 4 the EBs were placed onto gelatine coated culture dishes in CM10 medium for further differentiation with medium change every second day. After 1–4 days beating colonies arise in the cultures. The differentiation experiment was repeated three times to generate replicated biological samples for further data analysis.

### RNA extraction and microarray experiments

For the global transcriptional analysis, cells were sampled each day from day 0 to day 10 and total RNA was extracted daily during this time period using Ambion MagMaxTM-96 isolation kit according to the instructions from the manufacturer (Ambion, Inc., www.ambion.com), and quantified on NanoDrop ND-1000 (NanoDrop, www.nanodrop.com).

The quality of the RNA and cRNA, labelled by *in vitro* transcription, was verified using a 2100 Agilent Bioanalyzer. To measure the mRNA expression, fragmented cRNA was hybridized at 45°C for 16 hours to whole transcript Gene ST 1.0 arrays (Affymetrix, www.affymetrix.com). The microarrays were scanned on a GeneChip Scanner 3000 7G (Affymetrix). The raw expression data are available at ArrayExpress (http://www.ebi.ac.uk/microarray-as/ae/) with accession number E-MTAB-5219.

### Data analysis framework

**Fig 1:**
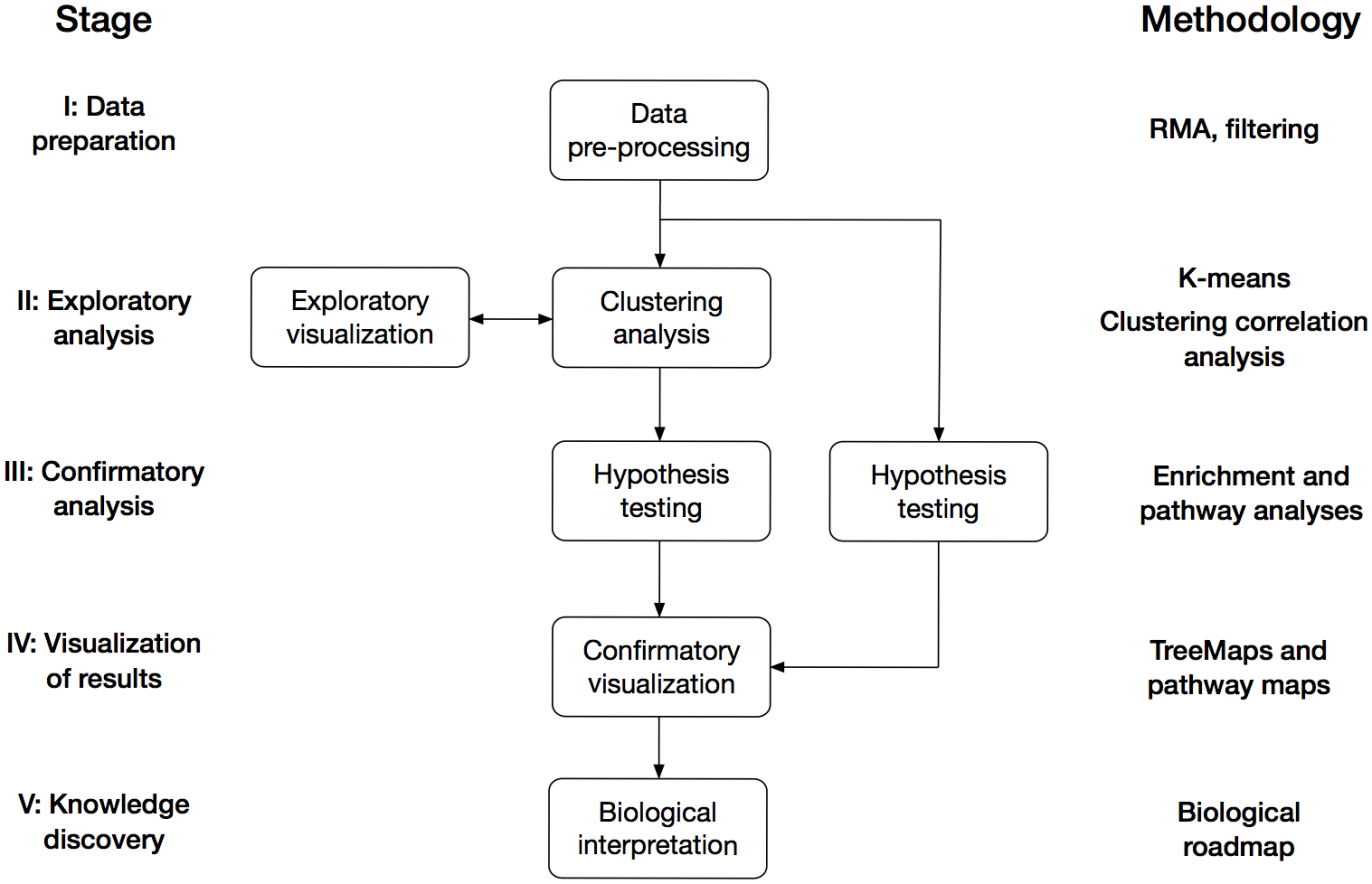
Data analysis framework. The figure illustrates the general analysis framework proposed in the study. The five stages of the framework are shown to the left and the steps within each stage are indicated by boxes. The specific methodology applied in the present study is shown to the right. Stage I: Data preparation. The raw microarray dataset was normalized with RMA and genes with low-expression or small variation in expression were removed. Stage II: Exploratory analysis. The pre-processed dataset of 1,108 genes and 11 time points was subject to k-means clustering with k = 10 and Pearson correlation as distance measure (see section Stage I: data preparation for more details). Stage III: Confirmatory analysis. Enrichment analysis was carried out for the genes in each k-means cluster to identify enriched Gene Ontology terms and transcription factors. Pathway analysis was performed with SPIA to infer pathway activity at each time point. Stage IV: Visualization of results. Biological processes significantly enriched among the genes in each cluster were visualized with TreeMaps. In addition, the impact of gene expression changes on pathway activity was clarified with pathway maps. Stage V: Knowledge discovery. The biological findings were incorporated into a roadmap that captures the main biological events and regulators of early hPSC differentiation towards the mesoderm lineage.

The following paragraphs describe the analysis framework for BBD proposed in the present study. The framework consists of five consecutive stages and exemplifies what analysis or processing to carry out at each stage (Fig 1). The purpose of the framework is to systematize the analysis process, and the analysis methodologies applied at each stage should be adapted to research questions of interest and the type of data analyzed.

Stage I (data preparation) deals with data-specific pre-processing procedures such as normalization and features filtering. The aim of this stage is to prepare high-throughput data for downstream analysis and remove any bias and irrelevant features from the data. The aim of Stage II (exploratory data analysis) is to discover “hidden” structures in the data through clustering analysis, and interpret these structures by means of different visualization techniques (exploratory visualization). Clustering analysis is performed by applying an algorithm to the data to find hidden structures of biological interest. Exploratory visualization is carried out using different plots or graphs to inspect the clustering results and evaluate the relevance of clusters obtained. These may be carried out iteratively to optimize the clustering of the dataset analyzed. For example, different parameter settings within the clustering algorithm can have a dramatic impact on the obtained clusters and should therefore be carefully chosen.

The main data analysis is carried out in Stage III (confirmatory analysis), where different hypothesis tests are performed to identify statistically significant and biologically relevant results. Confirmatory analysis is carried out for both clustered sets of features as well as for all features that pass the pre-processing to infer biological interpretation of the separate clusters as well as to the whole dataset. This is followed by confirmatory visualization in Stage IV (visualization of results). This stage aims to present the statistically and biologically significant results in such a way that they can be interpreted and translated to useful biological knowledge in Stage V (knowledge discovery). Useful visualization techniques exist to incorporate e.g. p-values, fold changes, signaling pathway topology and biological annotation term relationships [22,23]. The methodologies applied to the data analysis framework in the present study are described in subsequent sections.

### Stage I: Data preparation

Raw intensity signals were extracted and normalized with the Expression Console v1.1.2 (Affymetrix) using the robust multichip average (RMA) method. Gene expression values were calculated from the normalized data by taking the mean of all biological replicates (A, B and C) for each time point 0 to 10. Multiple probes that mapped to the same gene were collapsed by calculating the mean value of those probes. Furthermore, the dataset was filtered to remove probes that (i) did not map to known genes, (ii) genes expressed close to background, and (iii) genes that show small or no variation in gene expression. Thus, all probes that lack official gene symbol, probes with a log_2_ expression below 5 for all time points, and probes with a coefficient of variation below 10% were removed. The resulting dataset of 1,108 genes and 11 time points was subsequently used for clustering analysis. The gene symbols of these genes are found in S1 File.

### Stage II: Exploratory data analysis

Clustering analysis was applied to identify sets of genes with a similar response in expression profile over time, as the hESCs underwent differentiation towards the mesoderm lineage. The k-means algorithm [20], which is frequently used for clustering gene expression data, was applied for this purpose. Briefly, the k-means algorithm partitions the data into *k* disjoint clusters, where every data point (e.g. gene) belongs to the cluster with the nearest mean (centroid). The centroids are initially given coordinates from randomly selected data points, followed by an iterative step where data points are assigned to the nearest centroid and the centroids are set to the mean of these new coordinates. The result is a cluster assignment vector *v* = [*c*_1_, …, *c*_*n*_]^*T*^ where each *c*_*i*_ ∈ {1, …, *k*} specifies the cluster of the *i*th data point.

**Fig 2:**
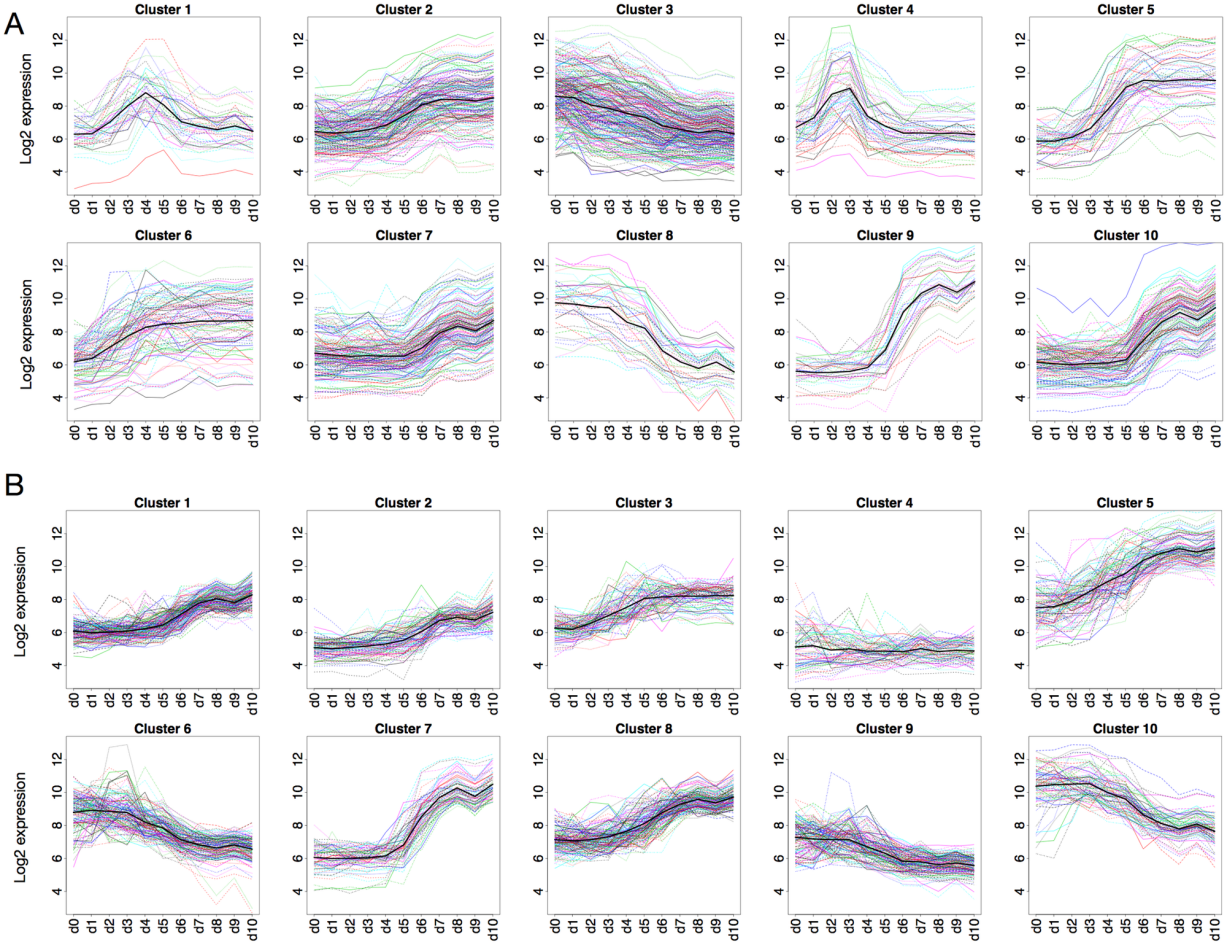
Cluster profiles of k-means clusters. (A) Pearson correlation and (B) Euclidean distance as distance measure. Each line represents a gene and color is used to distinguish individual genes in dense clusters. Euclidean clusters appear denser with genes being grouped together based on height along the Y-axis (gene expression). The clustering based on Pearson correlation appears sparser with genes grouped together based on the shape of expression profiles over time.

Clustering analysis was carried out in R using the *amap* package [24]. The distance measure defines how to calculate the distances between data points and cluster centroids. Pearson correlation was chosen because this gives clusters of genes with similar shape of the expression profiles, rather than clusters of genes with similar expression levels (as with Euclidean distance). The differences in cluster profiles for k-means run with Pearson and Euclidean are shown in Fig 2. Whereas Euclidean distance generated dense clusters (profiles are closer along the y-axis), Pearson correlation produced clusters of profiles with similar shape over time. For this study, clusters with correlated expression profiles were preferred since this suggests co-regulation of genes and hence similar biological function.

The number of clusters was set by investigating intra- and inter-cluster correlation for different values of *k*. Intra-cluster correlation was calculated as the mean correlation of genes in a cluster to the centroid of the cluster. Higher correlation within clusters implies that the cluster is homogenous, with highly similar expression profiles over the time points. Inter-cluster correlation was calculated as the average correlation between the cluster centroids and should be as low as possible. This is because high correlation between clusters implies that the clustering partition is fragmented, with the same profile shape in several clusters. The analysis revealed a principle increase in both intra- and inter-cluster correlation with larger values of *k*, which meant it was necessary to identify an acceptable trade-off. Based on this procedure, k-means clustering was applied using Pearson correlation coefficient as distance measure and the number of clusters was set to 10 (*k* = 10). This gave high intra-cluster correlation (0.92) and kept inter-cluster correlation below 0 (-0.01).

### Stage III: Confirmatory analysis

Annotation enrichment analysis (AEA) was performed to identify transcription factors and gene ontology annotation terms overrepresented for the genes in the k-means clusters. The analysis was carried out for each cluster separately using the Enrichr online tool (http://amp.pharm.mssm.edu/Enrichr/), which encompasses gene-set libraries based on a large number of annotation databases [25]. These databases contain information about which annotation terms (e.g. biological processes or pathways) are assigned to genes. In AEA, statistical tests are used to identify terms that occur in a list of genes with a frequency higher than expected by chance. These terms are defined as significantly enriched, and are assumed to be biologically relevant.

In the present study, transcription factors and Gene Ontology (GO) terms with a p-value < 0.05 following multiple testing correction were extracted. The Enrichr library for transcription factor regulation of genes is based on position-weighted matrices (PWMs) from JASPAR [26] and TRANSFAC [27]. The PWMs represent consensus-binding sites for each transcription factor and can be used to search for transcription factor binding sites. The gene ontology libraries in Enrichr are based on the Gene Ontology database [28].

To identify signaling pathways affected during the mesoderm differentiation of hESCs, Signaling Pathway Impact Analysis (SPIA) was carried out using the filtered dataset (1,108 genes). SPIA is implemented in the R package *spia* and can identify KEGG pathways that are significantly enriched in a set of genes [29]. In addition, gene expression fold changes and pathway topology are considered to infer impact on pathway activity. For example, if a set of genes are upregulated and activate downstream targets, the signal will propagate resulting in pathway activation. Based on this signal propagation, SPIA predicts if a pathway will be activated or inhibited as a consequence of input fold changes. Fold changes were calculated for all 1,108 genes by dividing gene expression at each time point (day 1 to 10) with expression at day 0 (using unlogged values). The calculated fold changes were then transformed to log_2_ scale and used as input to the SPIA algorithm.

### Stage IV: Visualization of results

In order to clarify the overarching biological meaning of annotation enrichment results for clustered genes, significantly enriched biological processes were visualized using REVIGO [23]. Annotation terms with p < 0.05 following multiple testing correction were considered significant. The results of the application of REVIGO are shown using TreeMaps. A TreeMap [30] is a visualization technique that depicts hierarchical data using nested rectangles, i.e. each branch of the tree is represented by a rectangle, which is then tiled with smaller rectangles showing sub-branches. Normally, the leaf nodes are coloured to show a separate dimension of the data and a leaf node’s rectangular area is proportional to another selected dimension of the data. In our application case, related GO terms are grouped into coloured branch rectangles with parent terms, while related and more specific terms are represented by sub-branches. The size of the rectangles is proportional to the statistical significance. The use of TreeMaps greatly simplifies the interpretation of biologically relevant processes and their various levels of abstraction, compared to long lists of partially redundant terms and their p-values.

The results from pathway perturbation analysis with SPIA were visualized as a relative pathway perturbation plot. The Y-axis of this plot (SPIA tA) represents the sum of all pathway fold changes at each time point compared to day 0. The sum is calculated following propagation of the fold change signal throughout the pathway based on its topology, and can be interpreted as a measure of relative pathway activity. It is based on this measure that SPIA infers if the pathway is either activated (tA > 0) or inhibited (tA < 0). To clarify the causes for pathway perturbation, the WNT signaling pathway was further visualized using a pathway map and a gene-transcription factor interaction network. The pathway map was downloaded from KEGG [31] and rendered with Pathview [22] using gene expression data for days 1 to 4. The gene-transcription factor network was created by including all genes in the WNT pathway present in the filtered dataset and all transcription factors in the Enrichr gene set library targeting at least one gene in the WNT pathway.

### Stage V: Knowledge discovery

Finally, the biological findings detected from confirmatory analysis and visualization were integrated into a summarizing overview, called a “biological roadmap” for early differentiation of hPSCs towards the mesoderm lineage. The roadmap seeks to capture the most significant biological changes in the cells over time, the genes involved in these changes, the transcription factors regulating those genes, and the signaling pathways activated at different time points. Drawing the roadmap requires detailed interpretation of the results by domain experts, to determine what finding correspond to state-of-the-art knowledge in the field, and which findings are novel. In the present study, results from the analysis framework assist expert-guided knowledge discovery, which confirms and extends current knowledge about early stem cell differentiation and may have implications for future research.

## Results

### Validation of selected mesoderm and cardiac markers during differentiation procedure

**Fig 3:**
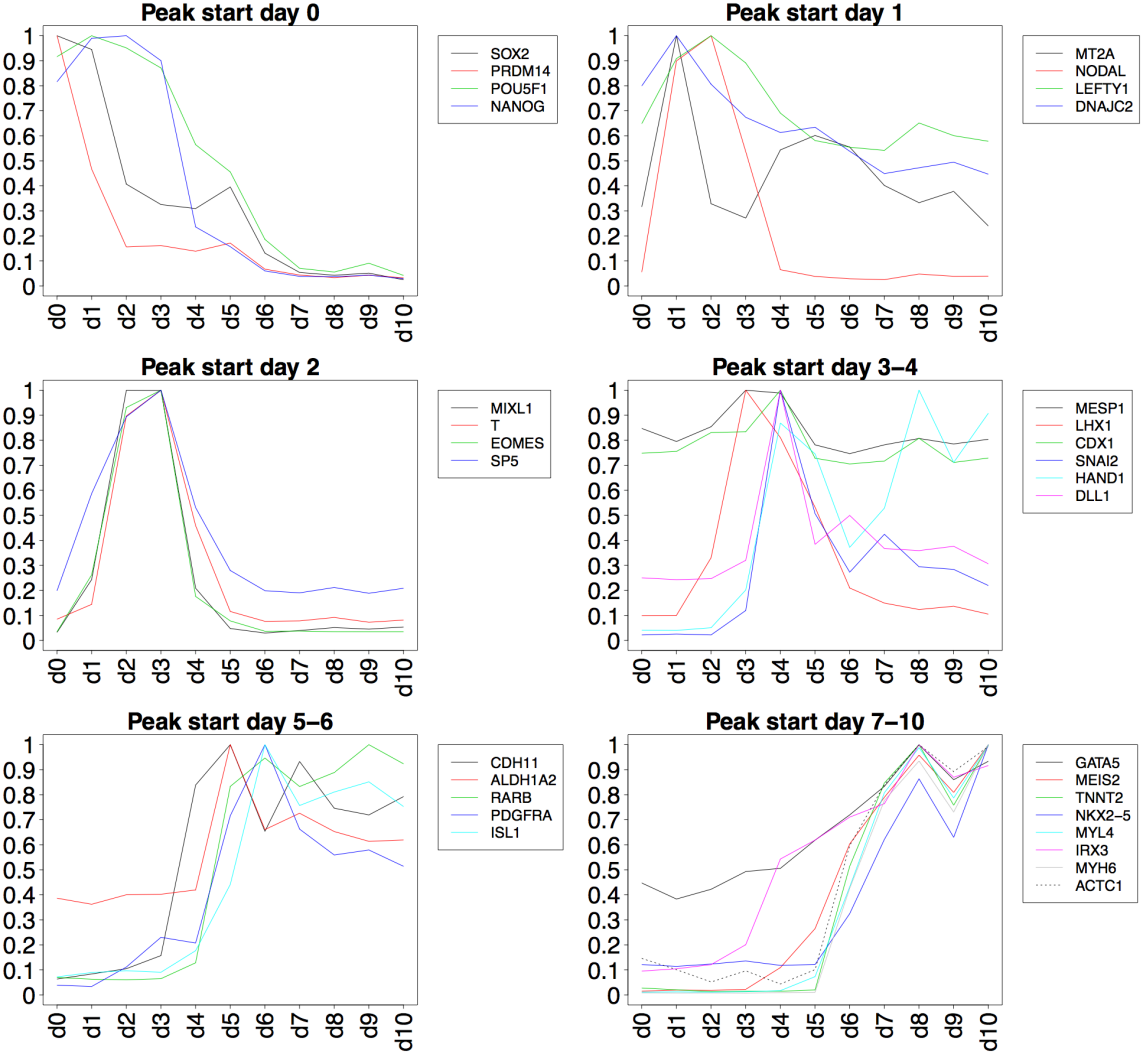
Temporal expression of selected mesoderm and cardiac markers. Expression profiles of known marker genes have been organized into six groups based on when peaks in expression are observed. Expression values have been normalized to the maximum expression value for each marker.

To verify that the cell culturing and differentiation protocol generated cells of the mesodermal lineage and cardiac specification, several known mesoderm and cardiac markers were monitored. Temporal expression of these markers in the microarray data were validated against cardiac induction results presented by Piccini *et al*. [9]. Expression profiles of the markers in the present study are presented in Fig 3, grouped by time point where a peak in expression is observed.

### Clustering analysis results

K-means clustering was applied to the filtered dataset with 1,108 genes and 11 time points, using *k* = 10 and Pearson correlation as distance measure. Contrary to the results produced by k-means with Euclidean distance, clusters based on Pearson correlation reveal different characteristic expression profiles of the genes (Fig 2). Therefore, clusters with Pearson correlation were used for subsequently analysis. A descriptive summary of these clusters is given in Table 1. The symbols of the genes present in each of the ten clusters can be found in S2 File. Clusters 1 and 4 show peaks early in the time series, day 4 and days 2–3, respectively. Remaining clusters show either upward or downward trends. The sharpest upward changes occur in clusters 5, 7, 9 and 10 where the vast majority of genes are activated simultaneously at a specific time point. The genes in cluster 8 show consistent downregulation across the time series, while clusters 2, 3 and 6 are more heterogeneous and do not display strong trends among the gene expression profiles.

**Table 1:** Descriptive summary of clusters.

Cluster	Size	Trend	Days
1	43	Peak	4
2	190	Upward	4–7
3	281	Downward	2–10
4	58	Peak	2–3
5	63	Upward	3–6
6	98	Upward	2–4
7	162	Upward	6–8
8	41	Downward	4–8
9	38	Upward	5–8
10	134	Upward	6–8

The clusters contain both well-known marker genes for the specific developmental stages as well as large groups of other genes not yet associated with mesoderm or cardiac development and their putative roles in mesoderm and early cardiac development remains to be elucidated. For example, cluster 4 contains 58 genes that show a distinct peak expression at day 2–3, which is the time point when the mesoderm initiation takes place. This cluster constitutes genes associated with the WNT signaling for example *T*, *NODAL*, *EOMES*, *MIXL1*, *DKK4*, but also typical mesendodermal marker genes such as *CER1*, *GSC*, and *KLF8* indicating that the cells are not yet fully primed towards the mesoderm lineage [32]. This is expected since mesoderm development is dependent on mesendoderm/endoderm signaling [33]. Cluster 5, which shows a peak expression at day 4 after onset of differentiation contains genes like *HAND1*, encoding a crucial cardiac regulatory protein that controls the balance between proliferation and differentiation in the developing heart [34] and is a marker of cardiac mesoderm [32], *MEIS1* and *MEIS2* where the latter has been reported to be involved in cardiac development [35], and *ISL1* a marker for cardiac mesoderm [32,36]. There are also clusters that show distinct upregulation at day 3 to 4, such as cluster 6 including *GATA4*, a cardiac transcription factor, *DUSP6*, a *MEF2A* target gene, *WNT5A* an activator of the Wnt/JNK pathway [37], and *DKK1* an inhibitors of WNT signaling [38,39]. In cluster 6 are also the *BMP2* and *BMP4* genes that are essential in cardiogenesis by inducing the expression of the cardiac transcription factors *NKX2-5* and *GATA4* [40].

Cluster 7 and cluster 10 show very similar expression profiles with a distinct upregulation at day 6 to 7. These clusters contain early cardiac markers such as *NKX2-5*, *MYL2*, *NPPA*, *NPPB*, *HCN1*, *MYH7*, *TNNC1*, and genes related to ion and Ca^2+^ handling such as *KCNIP2*, *KCNJ5*, *RYR2*, *SLN*, and *CASQ1*. Cluster 9 shows an even more distinct upregulation at a larger magnitude and includes for example *TBX5*, *TNNT2*, *VCAM1*, *PLN*, *MYH7*, *MYL3*, *MYL4*, *MYL7*, *BMP5*, *ACTA2*, *MEF2C*, *MYOCD*, and *WNT2* which all are highly associated to cardiac development.

### Enrichment analysis results

**Fig 4:**
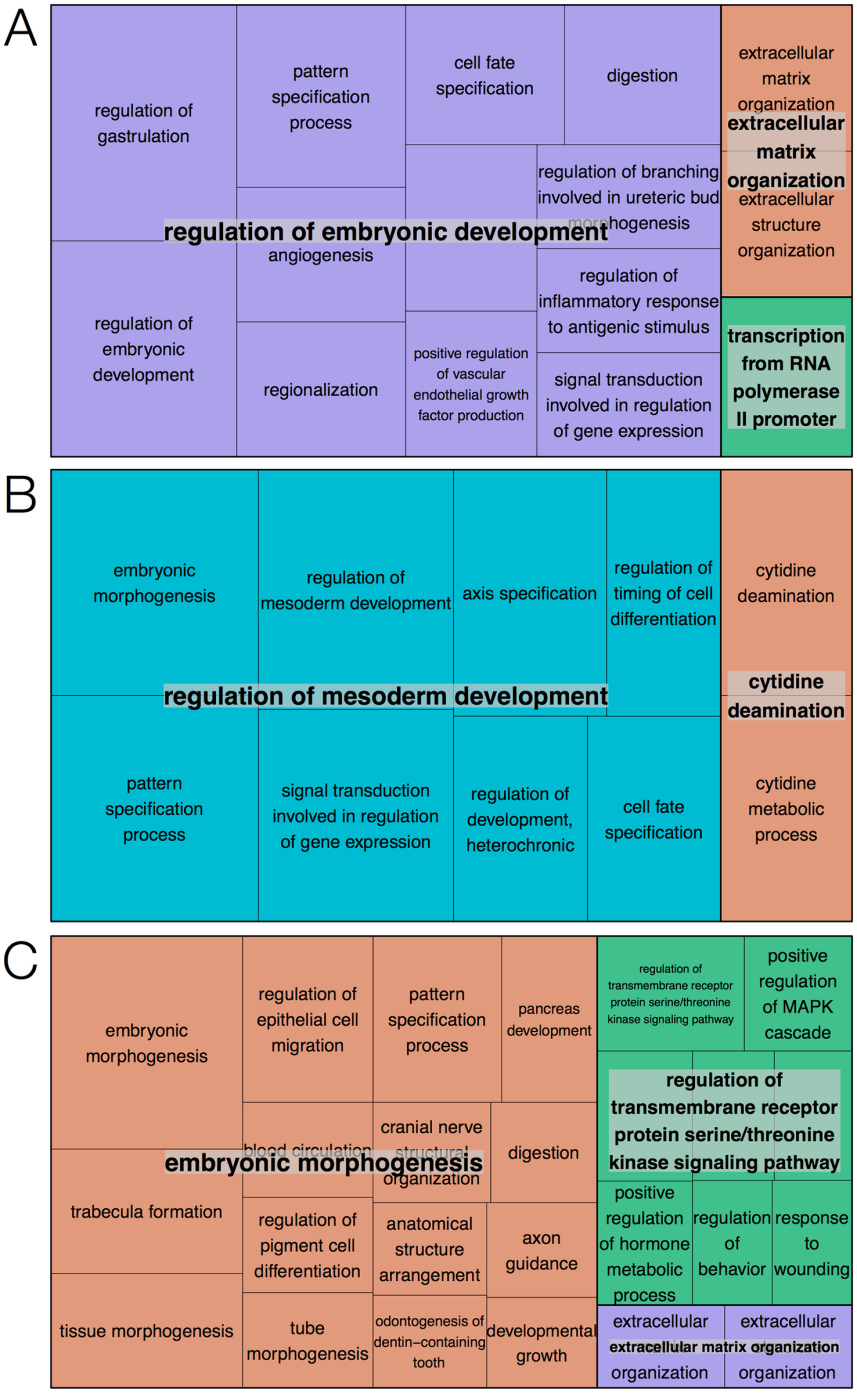
TreeMap visualization of significantly enriched biological processes. (A) Cluster 1, (B) cluster 4 and (C) cluster 5. Boxes represent terms and the size of the boxes reflects the significance of the corresponding p-value. Terms are grouped into overarching terms, which are visualized in different colors. The majority of terms for clusters 1, 4 and 5 are related to embryonic development.

**Fig 5:**
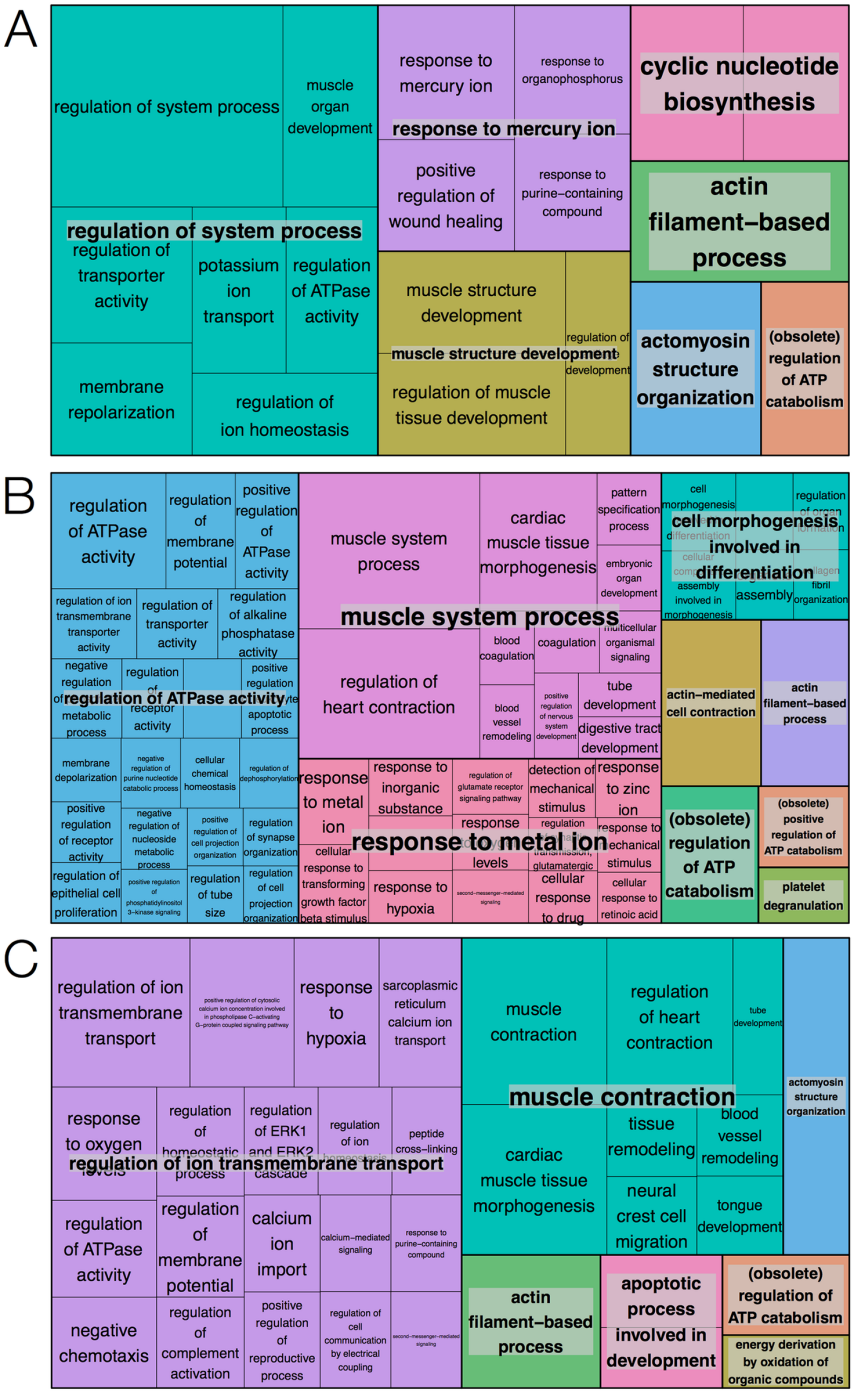
TreeMap visualization of significantly enriched biological processes. (A) Cluster 7, (B) cluster 9 and (C) cluster 10. Boxes represent terms and the size of boxes reflects the significance of the corresponding p-value. Terms are grouped into overarching terms, which are visualized in different colors. Many terms for clusters 7, 9 and 10 are related to muscle development and ion transport.

Annotation enrichment analysis was carried out with the genes in each of the ten k-means clusters. The analysis was performed with Enrichr and identified sets of significantly enriched Gene Ontology terms and transcription factors. To clarify the overarching biological roles of the set of genes in each of the clusters, biological process terms significant at p < 0.05 after multiple testing correction were visualized using REVIGO. The resulting TreeMaps highlight genes in clusters 1, 4 and 5 as mainly involved in embryonic development and morphogenesis. Similarly, genes in clusters 7, 9 and 10 were involved in several processes related to muscle development and ion transport. The TreeMaps for these clusters are shown in Figs 4 and 5, respectively. Only a few terms were significant for clusters 2 and 3, and genes in cluster 6 were involved in processes not related to mesoderm or cardiac development, e.g. kidney development and face morphogenesis. Biological processes enriched in cluster 8, with expression profiles showing a peak in the early stages of differentiation and rapid downregulation as cells maturate, were all related to stem cell maintenance. For these reasons, TreeMaps for clusters 2, 3, 6 and 8 were not included in the paper. Complete lists of enriched GO terms can be found in S3 File.

Transcription factors significantly enriched for genes in the specific clusters were identified to shed light on the regulatory mechanism behind the changes in gene expression profiles over time. The most significant results related to transcription factor enrichment are highlighted in Table 2, while complete lists of enriched transcription factors can be found in S4 File.

**Table 2:** Top five significant transcription factors for each cluster.

Cluster	Transcription factors
1	*CBEPA*, *JUN*
2	*FOXC1*, *POU2F2*, *TCF4*, *POU1F1*, *HMGA1*
3	*HINFP*, *FOXC1*, *STAT3*, *LEF1*, *RBPJ*
4	*ZBTB16*, *NFAT2*
5	*HMGA1*, *TEAD1*, *TBX5*, *BCL6*
6	*FOXL1*, *LEF1*, *HOXD9*, *BCL6*, *TCF4*
7	*TCF4*, *JUND*, *FOXC1*, *TBP*, *TEAD1*
8	
9	*YY1*, *POU1F1*, *CBEPB*, *FOXL1*, *IRF2*
10	*PGR*, *POU1F1*, *TCF4*, *GATA1*, *TBP*

### Pathway analysis results

**Fig 6:**
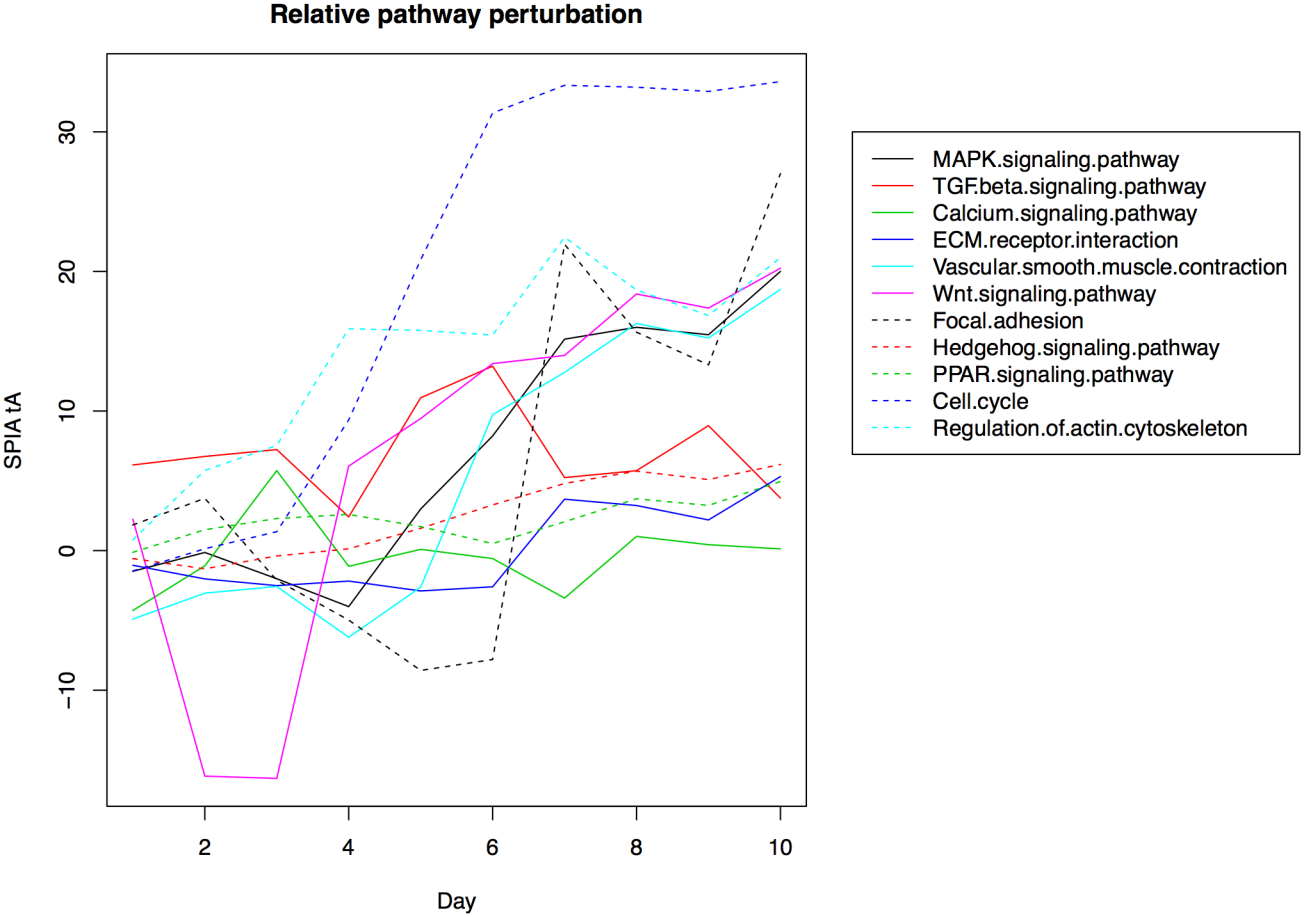
Relative pathway perturbation profiles. Each line represents a pathway identified as significant in the SPIA analysis. The Y-axis shows the *tA* score from SPIA, or total accumulated pathway perturbation. This is calculated as the sum of all pathway fold changes following propagation of fold changes based on pathway topology. A large positive number indicates that many genes in the pathway are upregulated, while a negative number indicates the opposite. Zero indicates that the pathway has the same activity as the baseline condition, i.e. day 0.

Pathway analysis was carried out with SPIA on all 1,108 genes that passed dataset filtering to identify significantly enriched pathways. SPIA also considers pathway topology to infer the perturbation in a pathway given a set of input fold changes. This analysis was performed for each time point of differentiation (day 1 to 10) compared to day 0 to obtain “pathway perturbation profiles” over the time series. The resulting profiles reveal what pathways become active during the differentiation process, and when (Fig 6).

A total of 24 pathways were significantly enriched by day 10 compared to day 0. Adjusted p-values and inferred pathway activity/status are given in Table 3. Some of the most significant pathways include cell cycle (activated), MAPK signaling (activated), TGF-β signaling (activated), and WNT signaling (activated). As illustrated in Fig 6, the cell cycle and WNT pathway show increased activity from day 4, while the MAPK pathway appears strongly activated from day 5. The TGF-β pathway appears to maintain activity throughout the time series.

**Table 3:** Significant signaling pathways identified by SPIA.

Name	Adj. p-value	Status
Dilated cardiomyopathy	1.30E-08	Inhibited
Cell cycle	2.24E-03	Activated
Pathways in cancer	2.24E-03	Activated
Retrograde endocannabinoid signaling	2.24E-03	Inhibited
MAPK signaling pathway	2.24E-03	Activated
TGF-beta signaling pathway	2.79E-03	Activated
Mineral absorption	2.79E-03	Inhibited
WNT signaling pathway	2.79E-03	Activated
Arrhythmogenic right ventricular cardiomyopathy (ARVC)	2.79E-03	Inhibited
GABAergic synapse	3.92E-03	Activated
ECM-receptor interaction	4.96E-03	Activated
Calcium signaling pathway	4.96E-03	Inhibited
Basal cell carcinoma	5.38E-03	Activated
Vascular smooth muscle contraction	5.83E-03	Activated
Neuroactive ligand-receptor interaction	8.25E-03	Activated
Melanoma	8.98E-03	Activated
Morphine addiction	1.02E-02	Activated
Focal adhesion	1.02E-02	Activated
HTLV-I infection	1.02E-02	Activated
Salivary secretion	1.11E-02	Inhibited
Hedgehog signaling pathway	1.61E-02	Activated
Melanogenesis	2.63E-02	Activated
PPAR signaling pathway	2.83E-02	Activated
Regulation of actin cytoskeleton	2.90E-02	Activated

**Fig 7:**
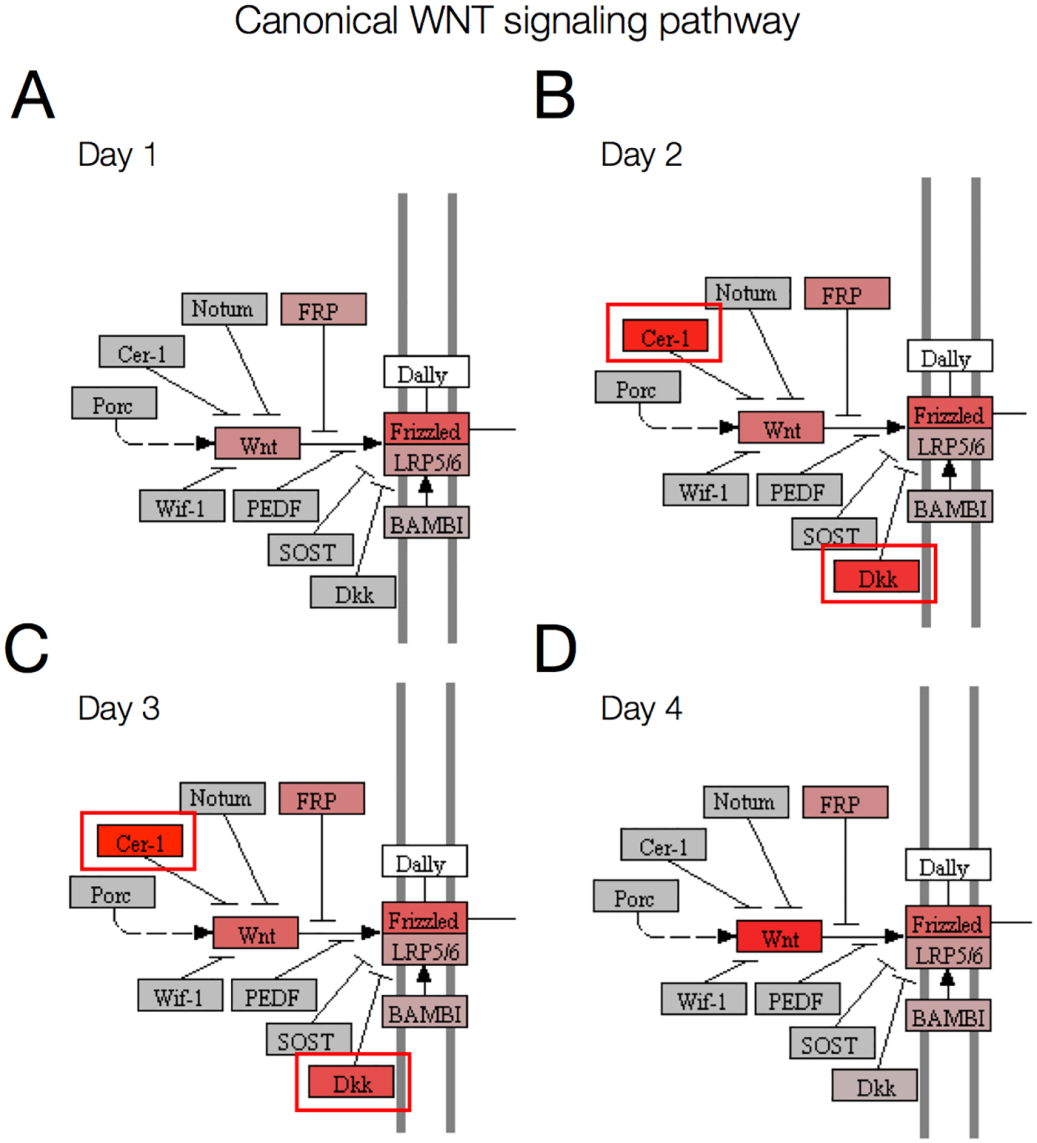
The canonical WNT signaling pathway in KEGG visualized with Pathview. Genes in the pathway are represented with rectangles. Gene expression is indicated with a color code, where red represents high expression and gray represents expression close to 0. (A) Expression at day 1, (B) expression at day 2, (C) expression at day 3, (D) expression at day 4. This pathway visualization reveals the activation of WNT inhibitors *CER1*, *DKK1* and *DKK4* at day 2 and 3 (indicated in red boxes).

**Fig 8:**
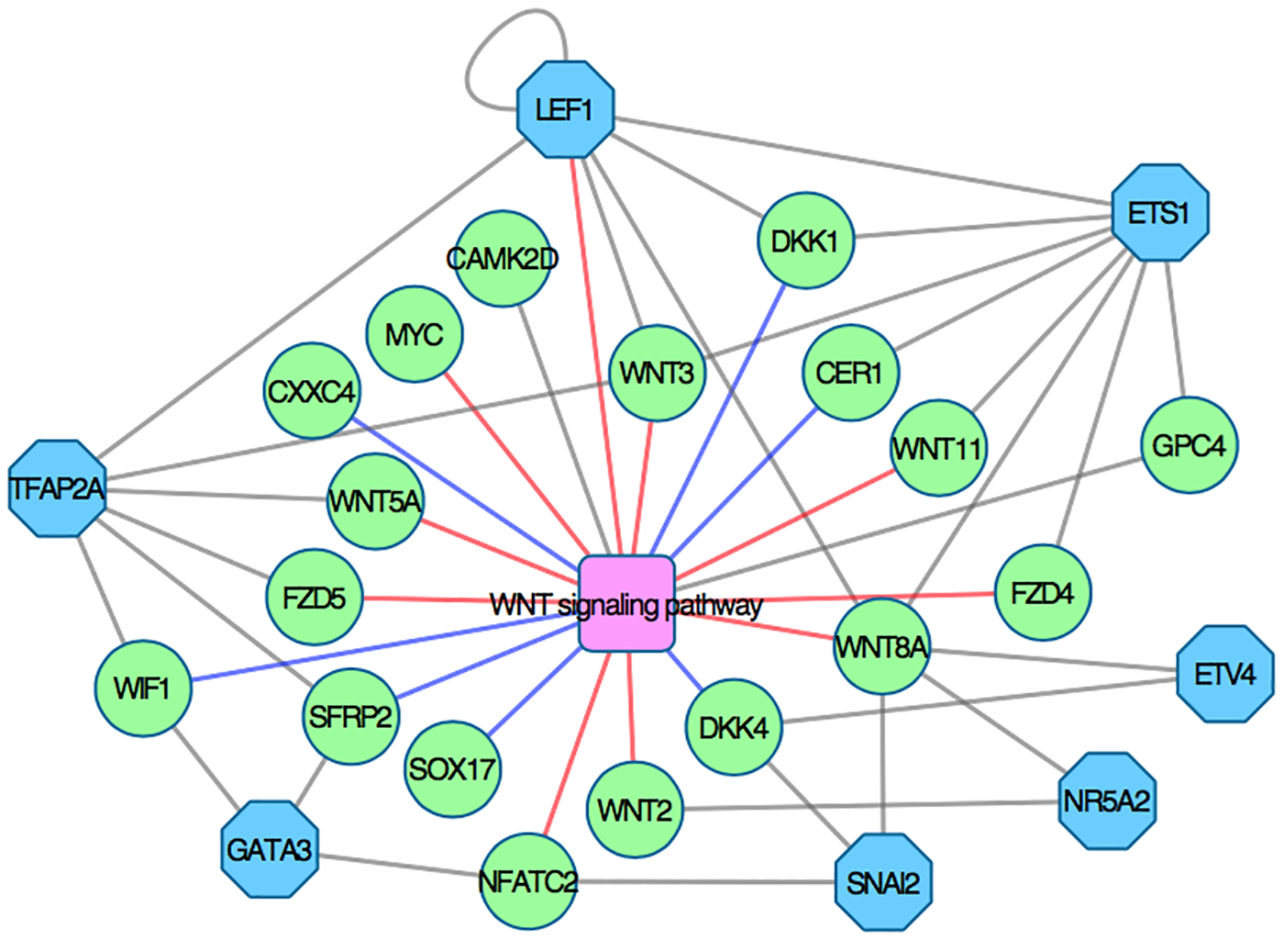
Gene-transcription factor interaction network. Genes in the WNT signaling pathway are represented by green circles and predicted transcription factors by blue octagons. When analyzed together with global transcriptome data, this network can shed light on the regulatory mechanisms behind pathway perturbation. Red edges indicate that genes activate the WNT pathway, while blue edges denote inhibitory genes.

To further clarify the mechanisms behind signaling pathways activation, pathway topology can be overlayed with expression data or fold changes. An example of this is provided in Fig 7, where the WNT signaling pathway in KEGG has been rendered with Pathview using gene expression data for day 1 to 4. This graph reveals that WNT inhibitors *CER1*, *DKK1* and *DKK4* are strongly activated at day 2 and 3. All three inhibitors are inactivated at day 4, resulting in WNT pathway activation (Fig 6). The transcription factor regulation of genes in the WNT signaling pathway is shown in Fig 8. Combined with global transcriptome data and pathway topology, this network can be used to study the regulatory mechanism behind pathway perturbation.

### A roadmap to mesoderm and early cardiac differentiation

**Fig 9:**
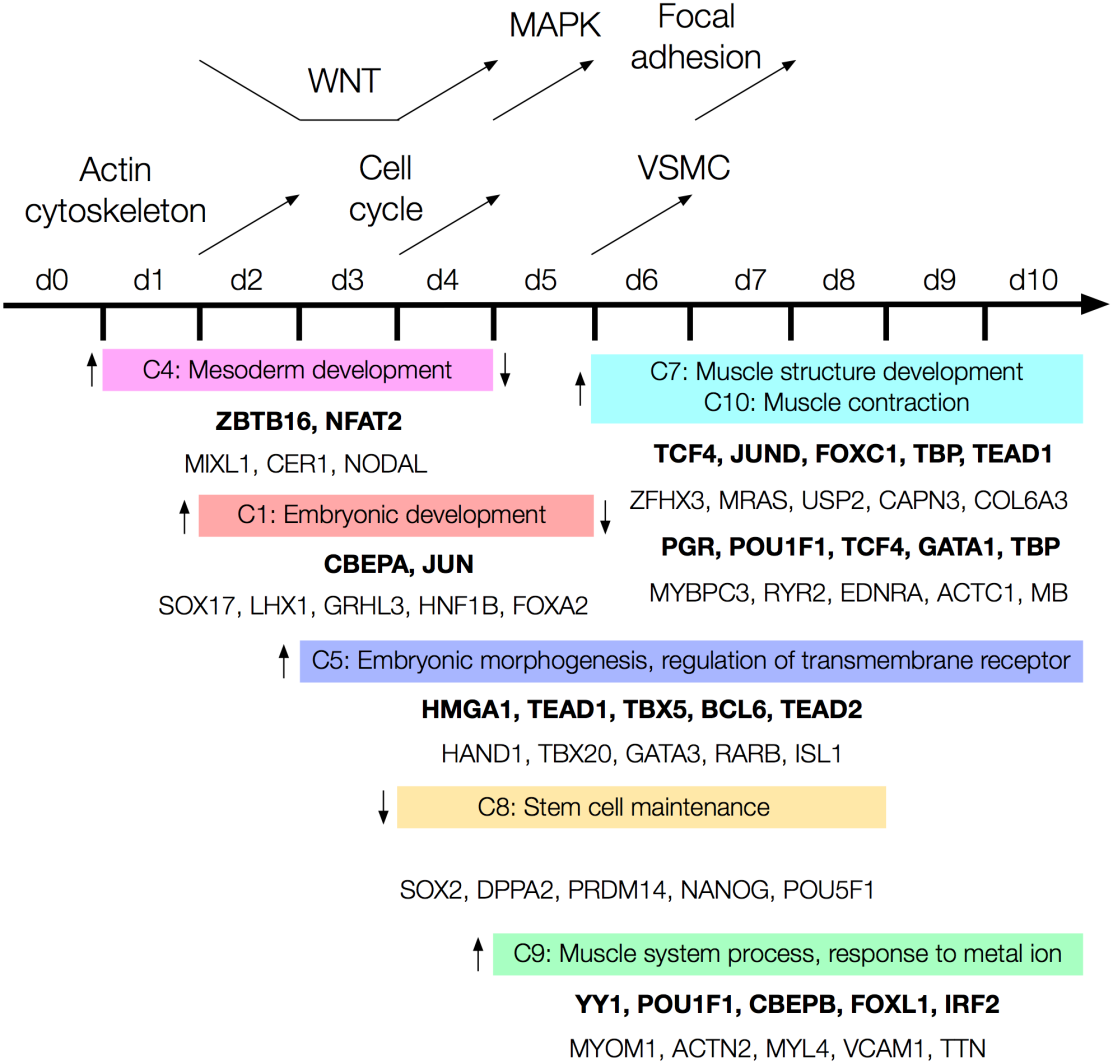
A roadmap for early differentiation of hPSCs towards the mesoderm lineage and cardiac specification. Time points are shown along the black horizontal line. Enrichment analysis results for clusters of genes are shown below, where each colored bar contains one/two representative terms from the TreeMap of a cluster (C1 denotes cluster 1). Transcription factors enriched for each cluster are shown in bold, and examples of genes in the cluster are given below the transcription factors. Vertical arrows beside the colored bars indicate changes in expression level of genes at different time points. For example, genes in cluster 4 are activated at day 1 and inactivated by day 4. Changes in signaling pathway activity are shown at the top, where arrows indicate at which time points different pathways are inhibited and activated.

The biological results from enrichment analysis of Gene Ontology, transcription factors and pathways in this study have been summarized into a roadmap for early differentiation of hESCs towards the mesoderm and cardiac lineages (Fig 9). Genes in clusters 1 and 4, showing peaks in activity at days 4 and 2–3 respectively, are clearly involved in embryonic and mesoderm development, including gastrulation. Genes in cluster 5 (activated at day 3) are involved in embryonic and cardiac tissue morphogenesis, and include several important cardiac related transcription factors such as *HAND1*, *TBX20*, *GATA3*, *RARB* and *ISL1*. Biological processes related to muscle and heart development are enriched in clusters 7, 9 and 10, which show sharp increases in activity at days 5 and 6. Genes in cluster 8 are strongly related to proliferation and stem cell maintenance and show consistent downregulation from day 4 to 8. Enrichment results were weak for clusters 2 and 3, which is why they have not been considered further. Cluster 6 shows several significant terms related to endoderm and ectoderm lineages, such as kidney development, epithelial cell differentiation and positive regulation of neurogenesis. These results may be attributed to heterogeneity in the cultured stem cell population, which includes cells along other differentiation trajectories.

Several pathways show increased activity throughout the time series (Fig 6). Regulation of actin cytoskeleton is activated during the first days of differentiation, while from day 4, the cell cycle and WNT signaling pathway become highly active. MAPK signaling, vascular smooth muscle contraction and focal adhesion show strong activation from day 5, 6 and 7 respectively. The TGF-beta pathway shows a consistent higher activity through the time series, while others e.g. calcium signaling, ECM receptor interaction, hedgehog signaling and PPAR signaling remain close to 0 (showing no perturbation compared to day 0).

## Discussion

### An analysis framework for biomedical big data

This study presents a data analysis framework to formalize the analysis protocol for large-scale omics data into five stages. The aim was to provide a frame of reference into which different analysis methodologies can be combined, depending on the research question addressed and the data analyzed. By following the framework, the analysis protocol becomes more transparent, facilitating reproducible research and tracking of data provenance. Furthermore, by combining different tools into a coherent process, the plethora of analysis workflows can be minimized and deeper knowledge discovery can be promoted. Our framework highlights the importance of in-depth exploratory data analysis as a precursor to any confirmatory analysis, similar to Tukey [14]. In the present study, the proposed analysis framework was applied to global transcriptomic time series data from early differentiation of hPSCs towards the mesoderm and early cardiac lineage. By integrating the results from the analysis, a comprehensive roadmap to mesoderm and early cardiac differentiation was inferred, revealing the temporal dynamics of key genes, transcription factors, biological processes and signaling pathways.

The data analysis framework developed in this study can be contrasted to other analysis protocols previously reported in the literature. Frequently, these analyses focus on certain aspects of the data, while omitting other potentially important facets. For example, while clustering is commonly applied to transcriptomics time series data, biological interpretation may focus only on certain genes, biological processes or signaling pathways [10–12,41,42]. In order to gain further biological insights from these high-throughput data, they should be explored from several perspectives. The data analysis framework presented in this paper takes a more holistic approach, combining several analysis strategies in one that facilitates comprehensive analysis and a deeper biological interpretation of the data. This is exemplified by the biological roadmap, which relies on multi-faceted biological analysis to capture the temporal dynamics of early hPSC differentiation.

The exploratory analysis carried out in this study demonstrates the importance of carefully choosing clustering algorithm parameters. By comparing k-means clusters obtained using either Euclidean or Pearson correlation as distance measure, the results from Pearson correlation showed higher biological relevance, as these clusters captured trends and patterns in expression profiles over time (Fig 2A). Euclidean distance, on the other hand, grouped genes together based on absolute expression level and failed to cluster expression profile peaks (Fig 2B). Clustering algorithms in different statistical software packages, for example *kmeans* [20] in R (The R Projects for Statistical Computing), may have Euclidean distance set as distance measure. This highlights the importance to adjust parameters based on the research questions addressed in the study, and shows how parameter choices affect the results obtained. Relying on default values may be inappropriate for many datasets, because biologically relevant structures in the data may be missed.

To promote interpretation of results and knowledge discovery, it is important to utilize intuitive visualization techniques that facilitate comprehension. A well-known problem with annotation enrichment analysis, particularly for Gene Ontology, is that it generates long lists of partially redundant terms, with various levels of specificity of biological meaning. These lists can be prohibitive to interpretation because of the amount of manual work that is required, and it can be difficult to overview the most significant results. Therefore, the proposed framework incorporated TreeMaps for collapsing related GO terms into parent terms and representing their relative statistical significance. TreeMap visualization greatly simplified interpretation of the AEA results in the present study, since the lists ranging from 18 to 242 enriched terms could be represented as TreeMaps with only one to nine parent terms. For example, 52 biological process terms were enriched for the genes in cluster 8 (showing consistent downregulating across time). REVIGO reduced these terms into a TreeMap with a single parent term: stem cell maintenance. Another powerful visualization technique for interpreting the impact of gene expression changes on pathways is to draw pathway maps overlaid with e.g. expression or fold change data. This incorporates gene expression changes in a cellular context and provides a way to deduce the causes for changes in pathway activity.

### Advancing understanding of early mesoderm differentiation of hPSCs

Despite intrinsic studies of the differentiation of hPSCs through the mesoderm germ layer and further to the cardiac lineage made by several investigators over the last decade, a thorough understanding of how these complex developmental processes are controlled is still lacking. New fundamental discoveries are reported continuously that provide new clues about the early human cardiac development. These discoveries include new knowledge about the specification of undifferentiated cells towards the cardiac lineage, and molecular mechanisms underlying these specification events, reviewed in detail in [43]. It also comprises knowledge about the differentiation potential of early cardiac progenitor cells and how these can be primed towards the cardiac differentiation. The highly dynamic endogenous and exogenous signaling directing mesoderm and early cardiac specification is challenging to study and relevant human models systems are a prerequisite. Here hPSCs constitute powerful tools for studies of early developmental processes using human cells [43].

Results from this study, where our data analysis framework has been applied to extensive time series data from mesoderm and early cardiac differentiation of hESCs, sampled daily from onset of differentiation until day 10, provide important additional insights into these complex regulatory processes of early cardiac initiation in human cells. The unique cocktail of addition and withdrawal of signaling factors during the differentiation procedure demonstrate typical gene expression response, which verifies the importance of each of the extrinsic initiation factors for the cardiac induction.

The results from these analyses confirm established knowledge such as the importance of expression of typical BMP factors and WNT signaling for the specification of the early mesoderm lineage, but also bring new and unknown insights of additional molecular pathways that also may play crucial roles during cardiac development. In addition to the frequently reported WNT pathway, which is crucial for promoting early cardiac differentiation [44] our analysis also identified the TGF-β/Activin A signaling pathway, which is known to play key roles during cardiac specification [45,46], and several other typical cardiac associated pathways such as Regulation of actin cytoskeleton-, Vascular smooth muscle contraction-, and Calcium signaling pathways as significantly overrepresented in the list of genes that show large response between day 0 and day 10 in our time series data. Moreover, based on our data analysis, interesting activation patterns indicate critical roles for other molecular pathways during cardiac precursor development. For example, the MAPK pathway shows a distinct upregulation from day 5 to day 10 and the Focal adhesion pathway is substantially inhibited at day 5 to 6, and from day 7 to day 10 it is again highly activated (see Fig 6).

WNT signaling plays an essential role in development and differentiation and is known to be critical during cardiac development both in human and in other species. Heart development is initiated by the induction of precardiac mesoderm requiring the tightly and spatially controlled regulation of both the canonical and the noncanonical WNT signaling pathways [37,47,48]. Canonical signaling is suggested to be involved in retaining the cardiac precursors in a proliferative and precursor state, while the noncanonical signaling promotes the cardiac differentiation [37,48]. Later on, both canonical and noncanonical signaling in parallel regulate specific steps in the development of the cardiac compartments [37,47,48]. A transient inhibition of the WNT signaling during the early mesodermal stage is a prerequisite for normal cardiac development [37,47,48]. Interestingly, this critical WNT-inhibition is mimicked in our time series data as shown in the relative perturbation plot in Fig 6, where a distinct downregulation of WNT signaling is present at day 2 and day 3, followed by a rapid upregulation at day 4 and forward. To further explore the WNT-signaling activation/inhibition during our experimental setup we compared our preprocessed data of 1,108 highly responsive genes with all known genes in the WNT signaling pathway reported in the KEGG pathway database and in total 19 (13%) of the genes in the WNT pathway are among the highly responsive genes (S1 File). Among these genes are *DKK1* and *DKK4*, which are inhibitors of WNT signaling, and the definitive endoderm genes *SOX17* and *CER1*. These 19 genes where further analyzed with Enrichr to identify putative transcription factors that control the expression of WNT signaling in our differentiation and seven transcription factors (*LEF1*, *TFAP2A*, *GATA3*, *ETS1*, *ETV4*, *NR5A2*, and *SNA2*) were predicted as putative regulators of these set of genes (Fig 8).

### Conclusions and implications for future research

This paper presented an analysis framework for BBD and applied it to global transcriptomics time series data from hPSCs differentiation towards the mesoderm lineage. The aim was to provide a frame of reference into which different analysis methodologies can be combined, to promote comprehensive data analysis and deeper biological knowledge discovery. Exploratory data analysis plays a key role in the framework, by exposing the hidden structures in the data and thus providing a point of departure for subsequent confirmatory analysis. Furthermore, the application of visualization techniques such as TreeMaps greatly facilitates interpretation of analysis results and help domain experts identify key findings. Taken together the analysis framework provides powerful means for analysis and interpretation of BBD.

Application of the framework to the hPSCs transcriptomics time series data revealed several processes, regulators and signaling pathways known to play key roles in mesoderm and cardiac differentiation. This both confirms validity of cell culturing and differentiation protocols, and adds to the body of published literature by providing high-resolution temporal dynamics of early hPSC differentiation. Importantly, by compiling the most significant findings into a biological roadmap, researchers can obtain a quick overview of the differentiation process and use this as a starting point for deeper knowledge discovery and comparative analysis.

Although yet only applied to transcriptomics time series data in the present study, the analysis framework can readily be applied to other types of omics data (e.g. proteomics and methylomics) and study designs. The analysis methodologies applied can and should be adapted to the data analyzed and research question addressed. This makes the framework useful for future research in the field of stem cell differentiation and, more generally, BBD analytics. Application of the framework allows significant results to be identified from several aspects of the data and presented in intuitive ways. When combined with domain expert knowledge, results can be complied into biological roadmaps. Apart from summarizing significant results, roadmaps can also serve roles in communication of biological knowledge.

## Supporting information

S1 File Gene symbols. Text file containing the 1,108 gene symbols used for analysis.
(TXT)

S2 File Cluster gene symbols. Excel file containing gene symbols for every k-means cluster.
(XLSX)

S3 File Enriched GO terms. Excel file containing enriched GO terms for every cluster.
(XLSX)

S4 File Enriched TFs. Excel file containing enriched transcription factors for every cluster.
(XLSX)

## References

[1] Margolis R, Derr L, Dunn M, Huerta M, Larkin J, Sheehan J, et al The National Institutes of Health’s Big Data to Knowledge (BD2K) initiative: capitalizing on biomedical big data. J Am Med Informatics Assoc. 2014;21: 957–8. 10.1136/amiajnl-2014-002974 PMC4215061 25008006

[2] Bacardit J, Widera P, Lazzarini N, Krasnogor N. Hard Data Analytics Problems Make for Better Data Analysis Algorithms: Bioinformatics as an Example. Big data. 2014;2: 164–176. doi: 10.1089/big.2014.0023 25276500 10.1089/big.2014.0023 PMC4174911

[3] Binder H, Blettner M. Big data in medical science—a biostatistical view. Dtsch Ärzteblatt Int. 2015;112: 137–42. 10.3238/arztebl.2015.0137 PMC4381554 25797506

[4] Ho P-J, Yen M-L, Yet S-F, Yen BL. Current Applications of Human Pluripotent Stem Cells: Possibilities and Challenges. Cell Transplant. 2012;21: 801–814. doi: 10.3727/096368911X627507 22449556 10.3727/096368911X627507

[5] Thies RS, Murry CE. The advancement of human pluripotent stem cell-derived therapies into the clinic. Development. 2015;142: 3077–3084. doi: 10.1242/dev.126482 26395136 10.1242/dev.126482 PMC6514400

[6] Li K, Kong Y, Zhang M, Xie F, Liu P, Xu S. Differentiation of pluripotent stem cells for regenerative medicine. Biochem Biophys Res Commun. 2016;471: 1–4. doi: 10.1016/j.bbrc.2016.01.182 26851367 10.1016/j.bbrc.2016.01.182

[7] Han D, Choi MR, Jung KH, Kim N, Kim SK, Chai JC, et al Global Transcriptome Profiling of Genes that Are Differentially Regulated During Differentiation of Mouse Embryonic Neural Stem Cells into Astrocytes. J Mol Neurosci. 2014;55: 109–125. doi: 10.1007/s12031-014-0382-8 25104607 10.1007/s12031-014-0382-8

[8] Nair R, Ngangan A V., Kemp ML, McDevitt TC. Gene Expression Signatures of Extracellular Matrix and Growth Factors during Embryonic Stem Cell Differentiation. PLoS One. 2012;7: e42580 doi: 10.1371/journal.pone.0042580 23077480 10.1371/journal.pone.0042580 PMC3471908

[9] Piccini I, Araúzo-Bravo M, Seebohm G, Greber B. Functional high-resolution time-course expression analysis of human embryonic stem cells undergoing cardiac induction. Genomics Data. Stellenbosch University; 2016;10: 71–74. 10.1016/j.gdata.2016.09.007 PMC5048627 27722090

[10] van den Berg CW, Okawa S, Chuva de Sousa Lopes SM, van Iperen L, Passier R, Braam SR, et al Transcriptome of human foetal heart compared with cardiomyocytes from pluripotent stem cells. Development. 2015;142: 3231–3238. doi: 10.1242/dev.123810 26209647 10.1242/dev.123810

[11] Satish L, Krill-Burger JM, Gallo PH, Etages S Des, Liu F, Philips BJ, et al Expression analysis of human adipose-derived stem cells during in vitro differentiation to an adipocyte lineage. BMC Med Genomics. BMC Medical Genomics; 2015;8: 41 doi: 10.1186/s12920-015-0119-8 26205789 10.1186/s12920-015-0119-8 PMC4513754

[12] Yang Z, Hao J, Hu Z-M. MicroRNA expression profiles in human adipose-derived stem cells during chondrogenic differentiation. Int J Mol Med. 2015;35: 579–586. doi: 10.3892/ijmm.2014.2051 25543998 10.3892/ijmm.2014.2051 PMC4314422

[13] Holzinger A, Dehmer M, Jurisica I. Knowledge Discovery and interactive Data Mining in Bioinformatics—State-of-the-Art, future challenges and research directions. BMC Bioinformatics. 2014;15 Suppl 6: I1. 10.1186/1471-2105-15-S6-I1 PMC4140208 25078282

[14] Tukey JW. Exploratory Data Analysis. 1st ed Pearson; 1977.

[15] Fathi A, Hatami M, Hajihosseini V, Fattahi F, Kiani S, Baharvand H, et al Comprehensive gene expression analysis of human embryonic stem cells during differentiation into neural cells. PLoS One. 2011;6: e22856 doi: 10.1371/journal.pone.0022856 21829537 10.1371/journal.pone.0022856 PMC3145766

[16] Fahad A, Alshatri N, Tari Z, Alamri A, Khalil I, Zomaya A, et al A Survey of Clustering Algorithms for Big Data: Taxonomy & Empirical Analysis. IEEE Trans Emerg Top Comput. 2014;2: 1–1.

[17] Madeira SC, Oliveira AL. Biclustering algorithms for biological data analysis: a survey. IEEE/ACM Trans Comput Biol Bioinform. 2004;1: 24–45. doi: 10.1109/TCBB.2004.2 17048406 10.1109/TCBB.2004.2

[18] Xu R, Wunsch DC. Clustering algorithms in biomedical research: A review. IEEE Rev Biomed Eng. 2010;3: 120–154. doi: 10.1109/RBME.2010.2083647 22275205 10.1109/RBME.2010.2083647

[19] Pirim H, Ekşioğlu B, Perkins A, Yüceer C. Clustering of High Throughput Gene Expression Data. Comput Oper Res. 2012;39: 3046–3061. doi: 10.1016/j.cor.2012.03.008 23144527 10.1016/j.cor.2012.03.008 PMC3491664

[20] Hartigan JA, Wong MA. Algorithm AS 136: A k-means clustering algorithm. J R Stat Soc Ser C (Applied Stat. JSTOR; 1979;28: 100–108.

[21] Burridge PW, Anderson D, Priddle H, Barbadillo Muñoz MD, Chamberlain S, Allegrucci C, et al Improved human embryonic stem cell embryoid body homogeneity and cardiomyocyte differentiation from a novel V-96 plate aggregation system highlights interline variability. Stem Cells. 2007;25: 929–38. doi: 10.1634/stemcells.2006-0598 17185609 10.1634/stemcells.2006-0598

[22] Luo W, Brouwer C. Pathview: an R/Bioconductor package for pathway-based data integration and visualization. Bioinformatics. 2013;29: 1830–1. doi: 10.1093/bioinformatics/btt285 23740750 10.1093/bioinformatics/btt285 PMC3702256

[23] Supek F, Bošnjak M, Škunca N, Šmuc T. REVIGO summarizes and visualizes long lists of gene ontology terms. PLoS One. 2011;6: e21800 doi: 10.1371/journal.pone.0021800 21789182 10.1371/journal.pone.0021800 PMC3138752

[24] Antoine L. amap: Another Multidimensional Analysis Package. R package version 0.8–14. 2014. https://cran.r-project.org/package=amap.

[25] Chen EY, Tan CM, Kou Y, Duan Q, Wang Z, Meirelles GV, et al Enrichr: interactive and collaborative HTML5 gene list enrichment analysis tool. BMC Bioinformatics. 2013;14: 128 doi: 10.1186/1471-2105-14-128 23586463 10.1186/1471-2105-14-128 PMC3637064

[26] Sandelin A, Alkema W, Engström P, Wasserman WW, Lenhard B. JASPAR: an open-access database for eukaryotic transcription factor binding profiles. Nucleic Acids Res. 2004;32: D91–4. doi: 10.1093/nar/gkh012 14681366 10.1093/nar/gkh012 PMC308747

[27] Wingender E, Chen X, Hehl R, Karas H, Liebich I, Matys V, et al TRANSFAC: an integrated system for gene expression regulation. Nucleic Acids Res. 2000;28: 316–9. 10592259 10.1093/nar/28.1.316 PMC102445

[28] The Gene Ontology Consortium. The Gene Ontology (GO) database and informatics resource. Nucleic Acids Res. 2004;32: D258–D261. doi: 10.1093/nar/gkh036 14681407 10.1093/nar/gkh036 PMC308770

[29] Tarca AL, Draghici S, Khatri P, Hassan SS, Mittal P, Kim J-S, et al A novel signaling pathway impact analysis. Bioinformatics. 2009;25: 75–82. doi: 10.1093/bioinformatics/btn577 18990722 10.1093/bioinformatics/btn577 PMC2732297

[30] Shneiderman B. Tree visualization with tree-maps: 2-d space-filling approach. ACM Trans Graph. 1992;11: 92–99.

[31] Kanehisa M, Goto S. KEGG: Kyoto encyclopedia of genes and genomes. Nucleic Acids Res. 2000;28: 27–30. 10592173 10.1093/nar/28.1.27 PMC102409

[32] Van Vliet P, Wu SM, Zaffran S, Pucéat M. Early cardiac development: a view from stem cells to embryos. Cardiovasc Res. 2012;96: 352–62. doi: 10.1093/cvr/cvs270 22893679 10.1093/cvr/cvs270 PMC3500045

[33] Foley AC, Mercola M. Heart induction by Wnt antagonists depends on the homeodomain transcription factor Hex. Genes Dev. 2005;19: 387–96. doi: 10.1101/gad.1279405 15687261 10.1101/gad.1279405 PMC546516

[34] Risebro C a, Smart N, Dupays L, Breckenridge R, Mohun TJ, Riley PR. Hand1 regulates cardiomyocyte proliferation versus differentiation in the developing heart. Development. 2006;133: 4595–606. doi: 10.1242/dev.02625 17050624 10.1242/dev.02625

[35] Louw JJ, Corveleyn A, Jia Y, Hens G, Gewillig M, Devriendt K. MEIS2 involvement in cardiac development, cleft palate, and intellectual disability. Am J Med Genet A. 2015;167A: 1142–6. 25712757 10.1002/ajmg.a.36989

[36] Moretti A, Lam J, Evans SM, Laugwitz K-L. Biology of Isl1+ cardiac progenitor cells in development and disease. Cell Mol Life Sci. 2007;64: 674–82. 17380308 10.1007/s00018-007-6520-5 PMC11138427

[37] Gessert S, Kühl M. The multiple phases and faces of wnt signaling during cardiac differentiation and development. Circ Res. 2010;107: 186–99. doi: 10.1161/CIRCRESAHA.110.221531 20651295 10.1161/CIRCRESAHA.110.221531

[38] Zhang P, Li J, Tan Z, Wang C, Liu T, Chen L, et al Short-term BMP-4 treatment initiates mesoderm induction in human embryonic stem cells. Blood. 2008;111: 1933–1941. doi: 10.1182/blood-2007-02-074120 18042803 10.1182/blood-2007-02-074120

[39] Bisson JA, Mills B, Paul Helt J-C, Zwaka TP, Cohen ED. Wnt5a and Wnt11 inhibit the canonical Wnt pathway and promote cardiac progenitor development via the Caspase-dependent degradation of AKT. Dev Biol. Elsevier; 2015;398: 80–96. 10.1016/j.ydbio.2014.11.015 25482987

[40] Li F, Deng X, Zhou J, Yan P, Zhao E, Liu S. Characterization of human bone morphogenetic protein gene variants for possible roles in congenital heart disease. Mol Med Rep. 2016;14: 1459–1464. doi: 10.3892/mmr.2016.5428 27357418 10.3892/mmr.2016.5428 PMC4940093

[41] Kim DS, Lee MW, Yoo KH, Lee T-H, Kim HJ, Jang IK, et al Gene Expression Profiles of Human Adipose Tissue-Derived Mesenchymal Stem Cells Are Modified by Cell Culture Density. PLoS One. 2014;9: e83363 doi: 10.1371/journal.pone.0083363 24400072 10.1371/journal.pone.0083363 PMC3882209

[42] Klein D, Benchellal M, Kleff V, Jakob HG, Ergün S. Hox genes are involved in vascular wall-resident multipotent stem cell differentiation into smooth muscle cells. Sci Rep. 2013;3: 2178 doi: 10.1038/srep02178 24145756 10.1038/srep02178 PMC3804857

[43] Calderon D, Bardot E, Dubois N. Probing early heart development to instruct stem cell differentiation strategies. Dev Dyn. 2016;245: 1130–1144. doi: 10.1002/dvdy.24441 27580352 10.1002/dvdy.24441 PMC5193096

[44] Mazzotta S, Neves C, Bonner RJ, Bernardo AS, Docherty K, Hoppler S. Distinctive Roles of Canonical and Noncanonical Wnt Signaling in Human Embryonic Cardiomyocyte Development. Stem cell reports. The Authors; 2016;7: 764–776. 10.1016/j.stemcr.2016.08.008 PMC5063467 27641648

[45] Zhang M, Schulte JS, Heinick A, Piccini I, Rao J, Quaranta R, et al Universal cardiac induction of human pluripotent stem cells in two and three-dimensional formats: implications for in vitro maturation. Stem Cells. 2015;33: 1456–69. doi: 10.1002/stem.1964 25639979 10.1002/stem.1964

[46] Rao J, Pfeiffer MJ, Frank S, Adachi K, Piccini I, Quaranta R, et al Stepwise Clearance of Repressive Roadblocks Drives Cardiac Induction in Human ESCs. Cell Stem Cell. 2016;18: 341–53. doi: 10.1016/j.stem.2015.11.019 26748419 10.1016/j.stem.2015.11.019

[47] Deb A. Cell-cell interaction in the heart via Wnt/β-catenin pathway after cardiac injury. Cardiovasc Res. 2014;102: 214–23. doi: 10.1093/cvr/cvu054 24591151 10.1093/cvr/cvu054 PMC3989450

[48] Ruiz-Villalba A, Hoppler S, van den Hoff MJB. Wnt Signaling in the Heart Fields: Variations on a Common Theme. Dev Dyn. 2016;245: 294–306. doi: 10.1002/dvdy.24372 26638115 10.1002/dvdy.24372

